# Efficient sky-blue perovskite light-emitting diodes via photoluminescence enhancement

**DOI:** 10.1038/s41467-019-13580-w

**Published:** 2019-12-10

**Authors:** Qi Wang, Xiaoming Wang, Zhi Yang, Ninghao Zhou, Yehao Deng, Jingjing Zhao, Xun Xiao, Peter Rudd, Andrew Moran, Yanfa Yan, Jinsong Huang

**Affiliations:** 1 Department of Applied Physical Sciences, University of North Carolina at Chapel Hill, Chapel Hill, North Carolina 27599 USA; 2 Department of Physics and Astronomy and Wright Center for Photovoltaics Innovation and Commercialization, The University of Toledo, Toledo, Ohio 43606 USA; 3 Electronic Materials Research Laboratory, Key Laboratory of Education Ministry, International Center for Dielectric Research, Xi’an Jiaotong University, Xi’an, 710049 China; 4 Department of Chemistry, University of North Carolina at Chapel Hill, Chapel Hill, North Carolina 27599 USA

**Keywords:** Devices for energy harvesting, Electronic devices

## Abstract

The efficiencies of green and red perovskite light-emitting diodes (PeLEDs) have been increased close to their theoretical upper limit, while the efficiency of blue PeLEDs is lagging far behind. Here we report enhancing the efficiency of sky-blue PeLEDs by overcoming a major hurdle of low photoluminescence quantum efficiency in wide-bandgap perovskites. Blending phenylethylammonium chloride into cesium lead halide perovskites yields a mixture of two-dimensional and three-dimensional perovskites, which enhances photoluminescence quantum efficiency from 1.1% to 19.8%. Adding yttrium (III) chloride into the mixture further enhances photoluminescence quantum efficiency to 49.7%. Yttrium is found to incorporate into the three-dimensional perovskite grain, while it is still rich at grain boundaries and surfaces. The yttrium on grain surface increases the bandgap of grain shell, which confines the charge carriers inside grains for efficient radiative recombination. Record efficiencies of 11.0% and 4.8% were obtained in sky-blue and blue PeLEDs, respectively.

## Abstract (web summary)

Despite the rapid progress on perovskite light emitting diodes (PeLEDs), the efficiency of blue PeLEDs is lagging behind. Here Wang et al. employ yttrium (III) chloride additive to yield enhanced photoluminescence in the perovskite materials and thus record high device efficiencies for sky-blue and blue PeLEDs.

## Introduction

Perovskite light-emitting diodes (PeLEDs) have drawn tremendous research interest in recent years due to the attractive properties of metal halide perovskite materials such as earth abundance, solution process capability, large carrier mobility, and excellent color purity^1–12^. The versatile bandgap tuning of perovskite materials (ABX_3_) via composition tuning of A, B, and X sites makes them applicable in whole visible light range. The application of all inorganic cesium-based perovskite significantly enhances the material stability under electric field^13,14^. Very impressive progresses have been made in enhancing the efficiencies of perovskite LEDs in the past few years^3–12^. External quantum efficiencies (EQEs) approaching 20% have been reported for both green and red PeLEDs, which ravel those from optimized organic phosphorescence light-emitting diodes (LEDs)^3–9^. However, blue-emission PeLEDs, which are important for display and lighting applications, have efficiency much lower than green and red PeLEDs. The highest reported EQE for sky-blue PeLEDs is still <10%^15–18^. One convenient way to make blue-emission perovskite films is to incorporate chlorine (Cl) into Br-based perovskites, whereas one critical issue for the Cl:Br mixed or pure Cl-based perovskite films is their small photoluminescence quantum efficiency (PLQE) below 20%, which is much lower than the green- or red-emission perovskite films with a typical PLQE of 70^3–9,19,20^. As only about a quarter of light could be extracted out of PeLED devices made on glass substrates due to the mismatched refractive index^21^, the highest EQE for the blue devices will be limited to <5%, assuming perfect charge balance can be achieved in the devices. The exact reason for the low PLQEs in chlorinated perovskite films is not clear. One way used in previous studies to increase PLQE was adopting quantum dots or two-dimensional (2D) layered perovskites, in which strong quantum confinement could significantly improve the film PLQE to over 80%^22^. However, due to the inferior charge transport in these films, high EQE sky-blue PeLEDs are still rarely reported.

In this study, we report significantly enhanced PLQE of sky-blue-emission polycrystalline perovskite films from 1.1% to 49.7% by incorporating PEACl and YCl_3_ into three-dimensional (3D) CsPbBr_3_ perovskite films, where PEACl is phenylethylammonium chloride (C_6_H_5_C_2_H_4_NH_3_Cl) and YCl_3_ is Yttrium (III) chloride. The sky-blue PeLEDs with 2% YCl_3_ showed high EQE of 11.0% and maximum brightness of 9040 cd m^−2^. Blue PeLED with 4.8% EQE was obtained by adding 10% YCl_3_ in the perovskite film, with a device Commission Internationale de l’Eclairage (CIE) coordinate of (0.10, 0.13).

## Results

### Photoluminescence enhancement by YCl_3_ in perovskite films

**Fig. 1:**
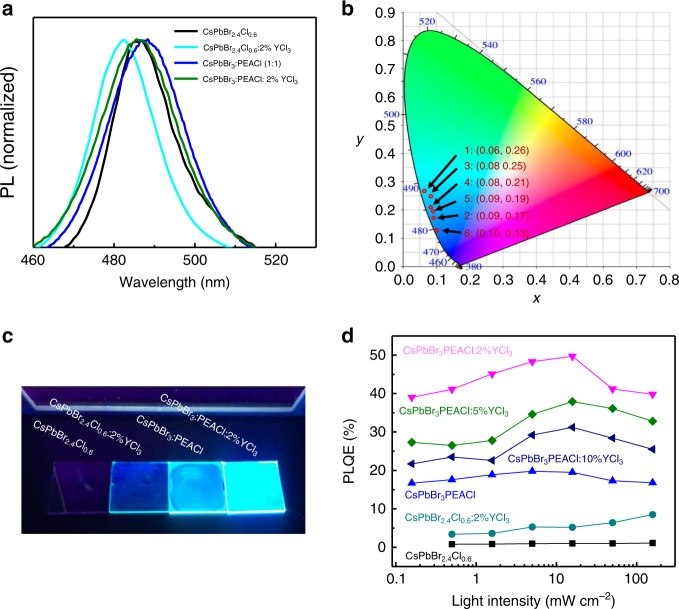
Photoluminescence enhancement by PEACl and YCl_3_ incorporation in perovskite films. **a** PL spectra of CsPbBr_2.4_Cl_0.6_, CsPbBr_2.4_Cl_0.6_:2%YCl_3_, CsPbBr_3_:PEACl (1:1), and CsPbBr_3_:PEACl:2%YCl_3_ films. **b** CIE coordinates of CsPbBr_2.4_Cl_0.6_ (1), CsPbBr_2.4_Cl_0.6_:2%YCl_3_ (2), CsPbBr_3_:PEACl (1:1) (3), and CsPbBr_3_:PEACl:2%YCl_3_ (4) films. CIE coordinates of CsPbBr_3_:PEACl:2%YCl_3_ (5) device and CsPbBr_3_:PEACl:10%YCl_3_ (6) device. **c** A photograph of the films under UV lamp. The composition of each film is labeled in the photograph. **d** Power-dependent PLQEs of the perovskite films with different compositions.

We fabricated and studied perovskite films with two different compositions in this study: 3D CsPbBr_2.4_Cl_0.6_ film and 2D–3D mixed CsPbBr_3_:PEACl film (molar ratio is 1:1 in precursor solution). Details about the film fabrication could be found in the Methods section. Chlorine was added in these films so that both films emit sky-blue light, with photoluminescence (PL) peaks at 486 nm for CsPbBr_2.4_Cl_0.6_ film and 487 nm for CsPbBr_3_:PEACl (1:1) film (Fig. 1a). The corresponding CIE coordinates are (0.06, 0.26) and (0.08, 0.25), respectively (Fig. 1b). Under the ultraviolet (UV) lamp, CsPbBr_3_:PEACl (1:1) film exhibits much brighter PL emission than the CsPbBr_2.4_Cl_0.6_ film (Fig.1c). Figure 1d shows PLQEs of CsPbBr_2.4_Cl_0.6_ film and CsPbBr_3_:PEACl (1:1) film under different incident laser intensities. Maximum PLQE of CsPbBr_3_:PEACl (1:1) film (19.8%) is much higher than that of CsPbBr_2.4_Cl_0.6_ film (1.1%). Phenylethylammonium halide incorporation in 3D perovskites usually introduces layered phases in the films, which has been demonstrated as an effective method to increase the PLQEs of green perovskite films^23,24^. Therefore, we focused on CsPbBr_3_:PEACl (1:1) perovskite in this study because of its higher PLQE compared with 3D CsPbBr_2.4_Cl_0.6_ film. PL spectra of perovskite films with different CsPbBr_3_:PEACl ratios are shown in Supplementary Fig. 1, which shows blue shift of PL peak from 522 nm for CsPbBr_3_ film to 487 nm for CsPbBr_3_:PEACl (1:1). Further increasing PEACl ratio in the films to CsPbBr_3_:PEACl (1:2) results in a much lower PL intensity and very broad emission band with multiple peaks, which might be from self-trapped exciton emission of different layered phases^25^. X-ray diffraction (XRD) study of the CsPbBr_3_:PEACl (1:2) film in Supplementary Fig. 2 clearly shows more layered phases are formed with the increased PEACl ratio in the films.

The PLQE of 19.8% in CsPbBr_3_:PEACl (1:1) film still limits the EQE of sky-blue PeLED to be <5%, considering a typical out-coupling efficiency around 20% in perovskite LEDs^21^. Encouragingly, we found that adding 2% YCl_3_ in the CsPbBr_3_:PEACl (1:1) film further increased PLQE to 49.7%, as shown in Fig. 1d. Here, the yttrium ratio is defined as the molar ratio of Y to Pb in the precursor solution. The photographs in Fig. 1c and Supplementary Fig. 3 clearly shows the PEACl:CsPbBr_3_ (1:1) films with YCl_3_ have much stronger PL emission than the film without YCl_3_. The maximum PLQEs of PEACl:CsPbBr_3_ (1:1) films with 2%, 5%, and 10% YCl_3_ are 49.7%, 37.9%, and 31.2%, respectively, which are much higher than the film without YCl_3_. Adding YCl_3_ in the PEACl:CsPbBr_3_ (1:1) film also blue shifts the PL peak (Supplementary Fig. 3). PEACl:CsPbBr_3_ (1:1) films with 2%, 5%, and 10% YCl_3_ exhibit PL peak at 485, 483, and 477 nm, respectively. The decreased PLQE in 5% and 10% YCl_3_ perovskite films compared with 2% YCl_3_ perovskite film might be caused by the formation of YCl_3_ clusters, which may hinder perovskite grain growth (Supplementary Fig. 4). Adding 2% YCl_3_ in the 3D CsPbBr_2.4_Cl_0.6_ film also significantly increases the film PLQE from 1.1% to 8.5%, as shown in Fig. 1d. The photographs in Supplementary Fig. 5 illustrate the PL of CsPbBr_3_ film was significantly improved by YBr_3_ treatment.

### Efficiency and stability of sky-blue PeLEDs with YCl_3_

**Fig. 2:**
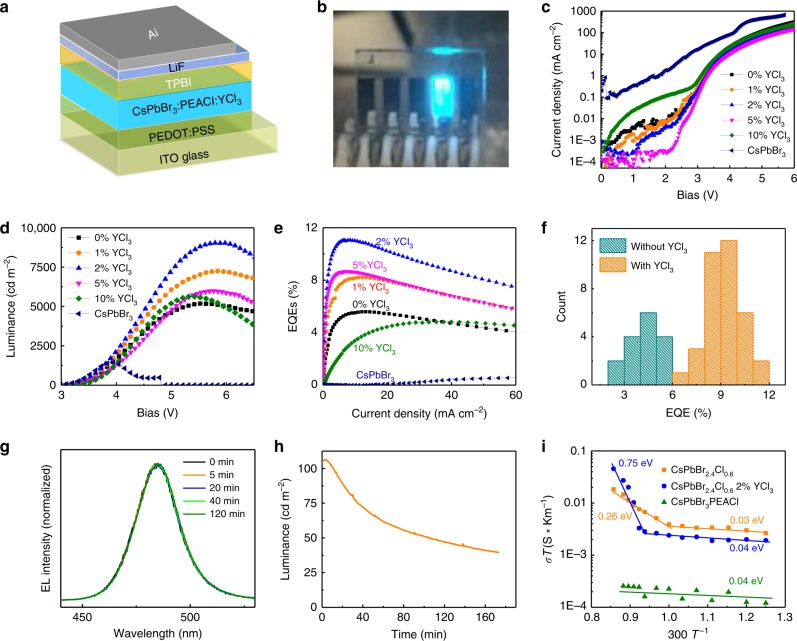
Efficiency and stability of sky-blue PeLEDs with YCl_3_. **a** Device structure of the PeLEDs. **b** A photograph of the fabricated PeLED showing sky-blue EL emission under bias. Current density-bias (**c**), luminance-bias (**d**), EQE-current density (**e**) curves of CsPbBr_3_:PEACl (1:1) devices with different ratios of YCl_3_. Current density-bias (**c**), luminance-bias (**d**), EQE-current density (**e**) curves of a CsPbBr_3_ perovskite LED. **f** Statistic EQEs of sky-blue PeLEDs with or without YCl_3_. **g** Electroluminescence spectrum stability test of a sky-blue PeLED with continuous bias of 3.2 V for 120 min. **h** Operational stability test of a sky-blue PeLED with initial luminance around 100 cd m^−2^. The stability test was conducted in N_2_ glovebox without device encapsulation. **i** Temperature-dependent conductivity measurements to reveal the ion migration activation energies of CsPbBr_2.4_Cl_0.6_, CsPbBr_2.4_Cl_0.6_ with 2% YCl_3_, and CsPbBr_3_:PEACl (1:1) films.

We fabricated PeLEDs with the device structure shown in Fig. 2a to evaluate the impact of the PLQE enhancement on device efficiency. PEACl:CsPbBr_3_ (1:1) perovskite films with different amount of YCl_3_ were fabricated between hole transporting layer (HTL) poly(3,4-ethylenedioxythiophene) polystyrene sulfonate (PEDOT:PSS), and electron transporting layer 2,2’,2”-(1,3,5-Benzinetriyl)- tris(1-phenyl-1-H-benzimidazole) (TPBI). A photo of an operating sky-blue PeLED is shown in Fig. 2b, which shows bright sky-blue emission. Figure 2c-e show current density (*J*)-bias (*V*), luminance (*B*)-bias (*V*), and EQEs curves of the PEACl:CsPbBr_3_ (1:1) devices with different ratios of YCl_3_. It was found that adding PEACl in the perovskite film improved the film morphology by forming pinhole-free and smooth film, as shown by the scanning electron microscopy (SEM) images in Supplementary Fig. 4. The pinhole free perovskite film reduced leakage current and improve efficiency in the device, as CsPbBr_3_ device showed large current leakage (Fig. 2c-e). The device with PEACl:CsPbBr_3_ (1:1) has an EQE of 5.6% and maximum brightness of 5183 cd m^−2^. Adding 2% YCl_3_ in the perovskite film significantly increases EQE and maximum luminance to 11.0% and 9040 cd m^−2^, respectively. Figure 2f shows statistic EQEs of PEACl:CsPbBr_3_ (1:1) devices without and with 2% YCl_3_, which clearly demonstrates the efficiency enhancement by adding YCl_3_ in the devices. Angular emission intensity of the PeLEDs follows a Lambertian profile, as shown in Supplementary Fig. 6. Further increasing the YCl_3_ ratio to 5% and 10% reduced device EQEs to 8.7% and 4.8%, respectively. The device with 10% YCl_3_ shows EL peak at 477 nm with a CIE coordinate of (0.10, 0.13), which comes to blue light region, as shown in Fig. 1b and Supplementary Fig. 7. The EQE variation of devices with different YCl_3_ ratios closely follows the trend of PLQE change in Fig. 1d, indicating the limiting factor for the efficiency of these sky-blue-emission PeLEDs is the PLQE of the perovskite films, whereas the electron and hole current has been well balanced through controlling the thickness of the charge transport layers in this work (Supplementary Figs. 8 and 9).

It has been widely reported that mixed-halide perovskites, such as CsPb(Br_0.5_Cl_0.5_)_3_, undergo a quick phase segregation under electric field, caused by charge-enhanced ion migration^22,26–29^. Such phase segregation can generate bromine-rich domains and chlorine-rich domains in CsPb(Br_0.5_Cl_0.5_)_3_ films, resulting in severe change of electroluminescent (EL) spectrum during the operation of PeLEDs^22,26–29^. We tested the EL spectrum and luminance stabilities of the most efficient sky-blue PeLEDs to evaluate whether such a phase segregation occurs in these mixed perovskite films. In striking contrast, we found the EL spectra are very stable for the PEACl:CsPbBr_3_ (1:1) LEDs with YCl_3_. Figure 2g shows the EL emission spectra of the sky-blue PeLED under constant bias of 3.2 V for 120 min. The device shows a stable EL emission with a peak at 485 nm. Supplementary Fig. 10 also shows negligible EL spectrum variation of the device under varied biases. The EL luminance stability of PeLEDs was also tested by fixing the applied voltage bias of 3.2 V, with an initial luminance around 100 cd m^−2^, as shown in Fig. 2h.

The significantly improved spectrum stability in PEACl:CsPbBr_3_ (1:1) films with 2% YCl_3_ compared with 3D perovskite materials was ascribed to the dramatically suppressed ion migration in these films, as phase segregation occurs via ion migration^28,30^. To verify it, we studied the activation energies of ionic conduction in both CsPbBr_2.4_Cl_0.6_ and PEACl:CsPbBr_3_ (1:1) films using an established method^31,32^. As shown in Fig. 2i, the ion conduction activation energy of 3D CsPbBr_2.4_Cl_0.6_ film in the dark is around 0.26 eV, which is consistent with previously reported values^33^. In striking contrast, we found that ionic conduction is negligible in the PEACl:CsPbBr_3_(1:1) film in the temperature range up to 350 K. The derived small activation energy of 0.04 eV can be assigned to electronic conduction. This indicates that the ion migration in the PEACl:CsPbBr_3_(1:1) films is significantly suppressed by the presence of layered perovskites, consistent with a previous study^34,35^. In addition, adding 2% YCl_3_ in 3D CsPbBr_2.4_Cl_0.6_ film also increased ion migration activation energy from 0.26 eV to 0.75 eV.

### Phase composition study in CsPbBr_3_:PEACl (1:1) film

**Fig. 3:**
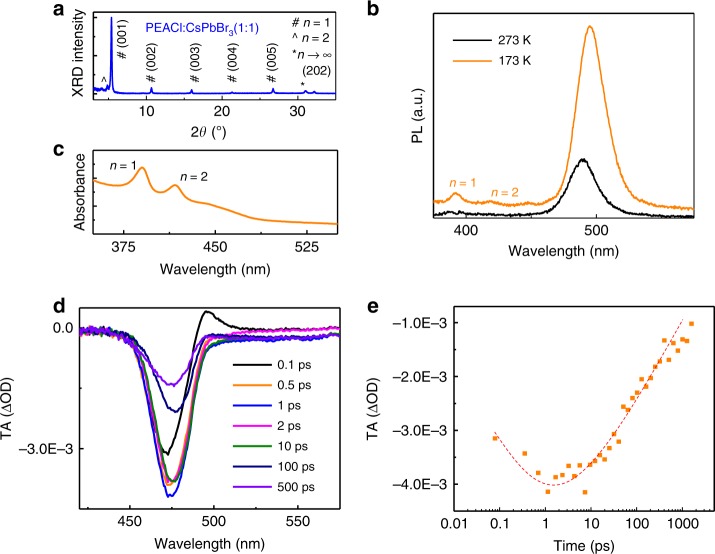
Phase composition study of CsPbBr_3_:PEACl (1:1) film and the function of layered phases. **a** XRD of CsPbBr_3_:PEACl (1:1) film. **b** PL spectra of CsPbBr_3_:PEACl (1:1) film at 273 K and 173 K. **c** Absorbance of CsPbBr_3_:PEACl (1:1) film. **d** Transient absorption (TA) spectra of CsPbBr_3_:PEACl (1:1) film at different timescales. **e** TA signal measured at a probe wavelength of 480 nm reveals distinct bleaching of the 3D phase in the CsPbBr_3_:PEACL (1:1) film.

To understand the role of PEACl, we studied phase composition of the PEACl:CsPbBr_3_ (1:1) film by performing XRD measurements. The XRD pattern of PEACl:CsPbBr_3_ (1:1) film in Fig.3a shows mixed phases of *n* = 1, *n* = 2, and *n* ≥ 3 perovskites, and each peak is indexed. Most XRD peaks of the PEACl:CsPbBr_3_ (1:1) film can be assigned to *n* = 1 phase, based on reported XRD pattern of (PEA)_2_PbBr_4_ single crystal^36^. The XRD peak intensity of the *n* = 1 phase is much stronger than the other phases, indicating *n* = 1 layered phase may prefer to lay down in parallel to the substrate. The Cl:Br ratio in *n* = 1 layered phase is estimated to be 1:4, based on the XRD and PL peak positions, as shown in Supplementary Figs. 11 and 12. The XRD peak at 4.3° is most likely from *n* = 2 phase, based on the analysis of lattice constants. The XRD diffraction peak of *n* ≥ 3 phase locates at a larger diffraction angle of 31.1° than (202) plane of CsPbBr_3_ (30.7°), whereas it is very close to the (202) plane of 3D perovskite with a composition of CsPbBr_2.4_Cl_0.6_, as shown in Supplementary Fig. 13. Hereinafter, we refer the *n* ≥ 3 phase in the PEACl:CsPbBr_3_ (1:1) film as a 3D phase. As shown in Fig. 3b, the PL spectrum from PEACl:CsPbBr_3_ (1:1) film at room temperature only has one emission peak around 489 nm. Reducing the temperature to 173 K leads to two additional peaks in the PL spectrum at 392 nm and 419 nm, which are most likely from *n* = 1 and *n* = 2 phases, respectively^37^. The PL emission around 490 nm in the PEACl:CsPbBr_3_ (1:1) perovskite films should be from the 3D phase with a composition approximately CsPbBr_2.4_Cl_0.6_. The absorbance spectrum of PEACl:CsPbBr_3_ (1:1) film in Fig. 3c also shows two obvious peaks at 390 nm and 418 nm, which are close to the PL emission peaks of *n* = 1 and *n* = 2 phases, respectively.

As the PL emission from the PEACl:CsPbBr_3_ (1:1) perovskite film at room temperature has only one peak from the 3D phase, there should be efficient energy transfer from layered perovskites to 3D perovskite. To verify it, we performed transient absorption measurement to study the charge-carrier dynamics in PEACl:CsPbBr_3_ (1:1) perovskite films. As shown in Fig. 3d, the bleach peak around 485 nm belongs to the 3D phase in the PEACl:CsPbBr_3_ (1:1) film. The bleach peak evolved in the first 2 ps after light excitation, suggesting a fast energy transfer from layered perovskites to 3D phase (Fig. 3e). After 2 ps, the energy funneling is complete, followed by charge recombination in 3D perovskite. The energy transfer from layered phases to 3D phase functions as an energy and carrier concentrator to enhance radiative recombination efficiency for higher PLQE, which has been reported in red-emission 2D–3D mixed perovskite films^38^. The promoted radiative recombination should give rise to higher PLQE observed in the PEACl:CsPbBr_3_ (1:1) perovskite films.

### Mechanism of PLQE enhancement by YCl_3_

**Fig. 4:**
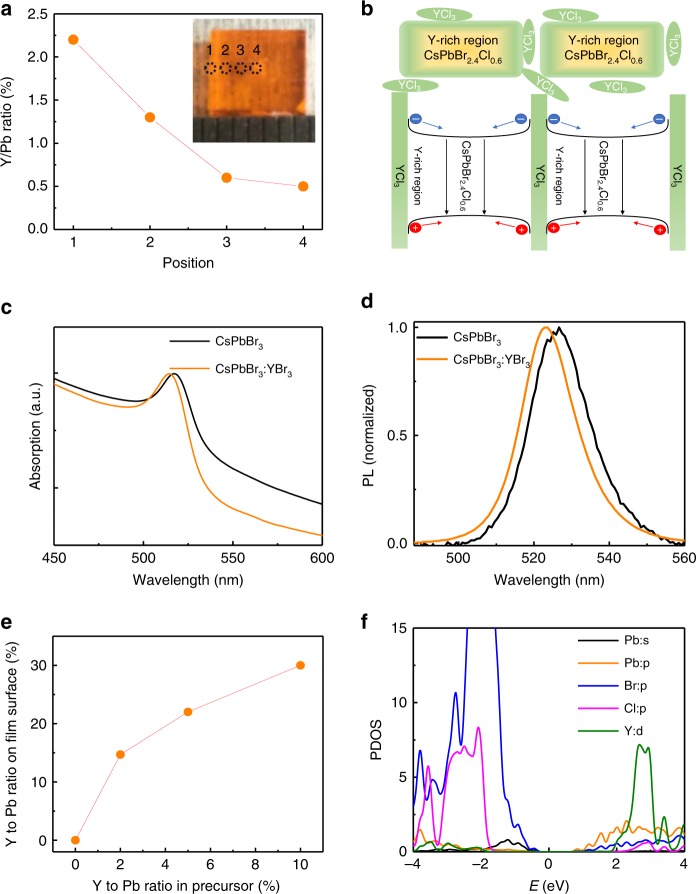
Mechanism of PLQE enhancement by YCl_3_. **a** ICP measurement results that show the Y/Pb ratios in the different locations of the crystal. The crystal (inset) was cleaved into thin pieces (1–4) and Y/Pb ratio of each piece was measured. **b** Schematic illustration of the yttrium gradient distribution in the CsPbBr_3_:PEACl (1:1) film and its function in increase the bandgap around the grain surface. Absorption (**c**) and PL (**d**) of CsPbBr_3_ films with or without YBr_3_. **e** XPS measurement results that show the Y/Pb ratios on the surface of CsPbBr_3_:PEACl (1:1) films with different YCl_3_ ratios of 0%, 2%, 5%, and 10%. The Y/Pb ratios on the film surface is higher than the corresponding Y/Pb ratios in the precursor solutions. **f** DFT-calculated PDOS of CsPbBr_3_ with YCl_3_.

We investigated the distribution of yttrium in the CsPbBr_2.4_Cl_0.6_ and PEACl:CsPbBr_3_ (1:1) films, to understand its functions in PLQE enhancement. We first examined whether yttrium ions incorporated into the 3D perovskite phase. We grew single crystals of CsPbBr_3_ with the presence of YCl_3_ in the solution. The Y distribution in the crystal was studied by inductively coupled plasma (ICP) mass spectrometry, which is a sensitive technique to detect trace metal ions as low as particle per quadrillion. The details about crystal growth can be found in the Methods section^39,40^. The single crystal was sliced into small pieces and the compositions of the crystal at different depth were measured. As shown in Fig. 4a, the Y/Pb ratio in the crystal center is 0.5%, demonstrating yttrium ions could incorporate into CsPbBr_3_ perovskite crystals. In addition, it was interesting to find the Y/Pb ratio gradually increase from the crystal center to surface, yielding an yttrium concentration gradient in the crystal. A gradient distribution of Y ions can be explained by the enhanced chemical pressure in the solution during the growth of crystal, because more Y ions excluded into solution during crystal growth increase its concentration in solution. Similar to single crystal growth, the larger amount of Y added in solution for thin film growth should also cause a concentration gradient, as illustrated in Fig. 4b. Absorption and PL study on thin films in Fig. 4c, d revealed the bandgap of CsPbBr_3_ was increased by Y incorporation. We also found a high density of yttrium on the perovskite film surface using surface-sensitive X-ray photoelectron spectroscopy (XPS) measurements. The XPS spectra of PEACl:CsPbBr_3_ (1:1) films with different ratios of YCl_3_ are shown in Supplementary Fig. 14. The Y 3*d*_3/2_ peak at 160.4 eV obviously increases with yttrium incorporation in the films. The Y/Pb ratios on the surfaces of the films derived from XPS spectra are shown in Fig. 4e. The Y/Pb ratios on the film surfaces are three to seven times higher than the corresponding Y/Pb ratios in the precursor solution, indicating that yttrium ions accumulate on the film surfaces or grain boundaries.

We examined possible mechanisms that explain the function of YCl_3_ in PLQE enhancement. Previous study showed organic ligands of layered perovskites have strong impact on the electron–phonon interaction and thus on PLQE of perovskites^41^. To find out possible impact of Y incorporation on the electron–phonon interaction, we studied the temperature-dependent PL full-width at half-maximum (FWHM) of PEACl:CsPbBr_3_(1:1) films with or without YCl_3_, which is a well-established method to derive an electron–phonon interaction^41–43^. As shown in Supplementary Fig. 15, the PL FWHMs of samples with and without YCl_3_ show the same trend at varied temperatures, which indicates that YCl_3_ does not notably change charge–phonon interaction strength. We did notice that addition of YCl_3_ reduced FWHM of PL spectra at all temperatures. This result implies YCl_3_ can reduce the nonradiative recombination centers in PEACl:CsPbBr_3_(1:1) films^41^, which is confirmed by more than two times longer PL decay lifetime after adding YCl_3_, as shown in Supplementary Fig. 16. Simulation was performed in CsPbBr_3_ perovskite incorporated with YCl_3_ to find out why Y^3+^ ions reduce nonradiative charge recombination. Based on the model, the way of Y^3+^ incorporation in CsPbBr_3_ is most likely by replacing Pb^2+^ and simultaneously forming a Cs^+^ vacancy to compensate the charge difference. As shown in Fig.4f, density function theory calculation reveals Y^3+^ incorporation increases the bandgap of perovskite, as Y 4*d* level is well above the Pb 6*p* states and the Cl 3*p* states are slightly lower than Br 3*p* states. Therefore, the yttrium incorporated region at the shell of the grains impose an energy barrier, which confines charge carriers inside the grains, as is illustrated in Fig. 4b. This charge confinement minimizes nonradiative recombination that generally occurs at grain boundaries and surfaces due to local large density of defects.

## Discussion

In conclusion, we demonstrated sky-blue PeLED with EQE of 11% and blue PeLED with EQE of 4.8%, by overcoming the limitation from the low PLQE of wide-bandgap perovskites. The combination of layered perovskite formation and YCl_3_ incorporation enhances the PLQE from 1.1% to 49.7%, which also enhance the material stability by suppressing ion migration. This study reveals that the low PLQE in most Cl:Br-based perovskites films limits the LED efficiency. Further research to improve the PLQE of wide-bandgap perovskite films to 100% should increase the EQE of sky-blue-emission PeLEDs to over 20%.

## Methods

### Material and solution preparation

PEACl was synthesized by reacting equal molar of phenethylamine (Sigma) with hydrochloric acid solution (Alfa Aesar 37%) under nitrogen at ice bath for 2 h with stirring. After reaction, the white precipitate PEACl was recovered by rotary evaporation at 80 °C and recrystallized by diethyl ether for three times. The PEACl powders were finally collected and dried at room temperature in a vacuum oven for 24 h. CsPb, PbBr, and YCl_3_ were purchased from Sigma Aldrich and TPBI was purchased from Lemtec. The perovskite precursor solution was prepared by mixing CsPbBr_3_ solution, PEACl solution, and YCl_3_ solution with desired ratios. CsPbBr_3_ solution (0.25 M) was prepared by mixing 106 mg CsBr and 183.5 mg PbBr_2_ in 2 mL dimethylsulfoxide (DMSO). The CsPbBr_3_ solution was filtered before using. PEACl solution (1 M) was prepared by mixing 158 mg PEACl in 1 mL DMSO. YCl_3_ solution (0.25 M) was prepared by mixing 49 mg YCl_3_ in 1 mL DMSO.

### Perovskite LED fabrication

The indium tin oxide (ITO)-coated glass substrates were sequentially cleaned in detergent, distilled water, acetone, and isopropanol by sonication. The cleaned substrates were treated in UV ozone for 15 min. The, PEDOT:PSS (AI 4083) aqueous solution was spin-coated at 3500 round per minute (RPM) for 40 s and baked at 125 °C for 30 min in ambient air. After that, the substrates were transferred into a nitrogen-filled glovebox and perovskite solution was spin-coated on top of substrate at 4000 RPM for 40 s. Five hundred microliters of chloroform was poured onto perovskite film 20 s after the spin coating started. The perovskite films were annealed at 90 °C for 20 min. Finally, the fabrication of PeLEDs was completed by thermally evaporating TPBI (40 nm), LiF (<1 nm), and Al (100 nm) electrode. The device area was 0.10 cm^2^ as defined by the overlapping area of the ITO and Al electrode.

### Perovskite film and device characterizations

Absorption and PL spectra were measured by Evolution 201 UV-Visible Spectrophotometer and iHR320 Photoluminescence Spectroscopy, respectively. SEM image was measured by FEI Helios 600 system. XPS was measured by Kratos Axis Ultra DLD X-ray Photoelectron Spectrometer. The surface compositions of samples were analyzed by the Processing software on the system. The current (*J*)–voltage (*V*)–luminance characterizations of the devices were performed in N_2_ glovebox without encapsulation. A Keithley 2400 source meter was used to measure the *J*–*V* data from 0 V to 7 V with a voltage scanning speed around 0.2 V s^−1^. The luminance of the device was recorded simultaneously by Konica Minolta, LS-160, or a calibrated silicon photodiode (Hamamatsu, S2387 1010 R) with an area of 10 mm × 10 mm. The photodiode was placed on top of the LED in close contact to collect light. The EQE was calculated using Lambertian profile and the obtained electroluminescence spectrum.

*Photoluminescence quantum efficiencies* were measured by following previous established method^44^. An integrating sphere (Labsphere QE sphere) was connected with a PL spectrometer (Ocean Optics QEpro) by an optical fiber. A continuous wave 403 nm laser was used to excite the sample. The incident laser intensity was changed by neutral density filters and the intensity was measured by a power meter (Newport 843R). The samples for PLQE measurement were encapsulated and the measurement was performed in air at room temperature. The PLQE results were confirmed by our collaborator at Xi’an Jiao Tong University (Supplementary Fig. 17). Edinburgh FLS980 fluorescence spectrometer system was used in collaborator’s PLQE measurement. The Edinburgh FLS980 fluorescence spectrometer has an integrating sphere with a diameter of 150 mm. The excitation light was a monochromatic 365 nm light and the intensity is 0.5 mW cm^−2^.

*ICP mass spectrometry* was carried out using a Thermo Element XR-Optical laser ablation source with a double focusing magnet sector. Standards were prepared from THERMO-54 and MSPB calibration standards from Inorganic Ventures and calibration curves were produced for yttrium (Y) to lead (Pb) from 25 parts per trillion to 500 parts per billion. CsPbBr_3_ with 2% YCl_3_ crystals were grown by inverse-temperature crystallization method, which has been widely used to grow high-quality perovskite crystals. Four surfaces of the crystals polished by sand paper to expose the crystal internal. Then the crystal was cleaved into about 1 mm thin piece by using operation blades. To prevent contamination, each sample was sealed in different vials and the blade was carefully cleaned after each cutting. The samples were then dissolved in 1 mol% HNO_3_ (prepared from Fisher Chemical Nitric Acid TraceMetal Grade and NERL 18 MΩ water). The solutions were then further diluted with 18 MΩ water to reach a concentration within the range of the calibration curve. Solutions were injected into the nebulizer and injected into the instrument for analysis. Each sample was followed by a washing period with 1 mol% HNO_3_ in 18 MΩ water before the next sample. Using the calibration curves produced, the ratio of yttrium (Y) to lead (Pb) was determined.

*Time-resolved photoluminescence (TRPL) measurement* was performed on a Horiba DeltaPro fluorescence lifetime system. The excitation was provided by a DeltaDiode (DD‐405) pulse laser diode with a wavelength of 404 nm. The TRPL curves were fitted to a monoexponential rate law:

1 $$y = A\ {\mathrm{exp}}\left( { - \frac{t}{\tau }} \right) + y_0$$

where *A* is the relative amplitudes and *τ* is the lifetimes. The samples for TRPL measurements were perovskite films deposited on glass substrates without HTL. The fluence of TRPL excitation laser and the generated carrier density were 2.0 nJ cm^−2^ and 4.07 × 10^14^ cm^−3^, respectively.

*Temperature-dependent conductivity measurement* for deriving ion migration activation energy was performed in a Lakeshore probe station under a vacuum of 10^−4^ pa^31,32^. The samples were placed on a copper plate with its temperature being controlled by a heater and injected liquid N_2_. A Keithley 2400 was used for applying voltage bias and measuring the current. The samples for conductivity measurement have lateral Au electrodes on perovskite film. Perovskite films were first fabricated on glass substrates by following the above-mentioned method. Then, 50 nm Au electrodes with spacing of 50 μm were thermally deposited using a mask. Ion activation energy can be derived by fitting the conductivity curve with Nernst–Einstein equation:

2 $$\sigma \left( T \right) = \frac{{\sigma _0}}{T}{\mathrm{exp}}\left( { - \frac{{E_{\mathrm{A}}}}{{k_{\mathrm{B}}T}}} \right)$$

where *σ* is the conductivity, *T* is the temperature, *E*_A_ is the activation energy, and *k*_B_ is the Boltzmann constant.

*Transient absorption experiment* was conducted with a 45 fs, 4 mJ, 800 nm Coherent Libra with a 1 kHz repetition rate. Single-frequency 400 nm pump pulses were generated by the second harmonic generation through a barium borate crystal. The remaining 800 nm component was filtered by two 400 nm dielectric mirrors. The pulse energies were controlled by a neutral density filter. Continuum probe pulses were generated in a sapphire window and relayed to the sample with reflective optics. The spot size of the probe was adjusted to match the 200 μm spot size of the pump. Signal detection was accomplished with a high-speed complementary metal-oxide semiconductor array detector that is synchronized to the laser system.

*Temperature-dependent PL spectrum measurement* was performed by using iHR320 Photoluminescence Spectroscopy and a portal LINKAM thermal stage. The FWHM of PL spectra was fitted by the following equation to derive an electron–phonon interaction strength ^41–43^:

3 $${\mathrm{\Gamma }}\left( T \right) = {\mathrm{\Gamma }}_0 + \frac{{{\mathrm{\Gamma }}_1}}{{{\mathrm{exp}}\left( {\frac{{\hbar \omega _1}}{{k_{\mathrm{B}}T}}} \right) - 1}}$$

where Γ_0_ is temperature-independent inhomogeneous broadening term, which arises from scattering due to disorder and imperfections. Γ_1_ represents the electron–phonon coupling strength, primarily contributed by longitudinal optical phonon scattering. *ω*_1_ is the homopolar phonon frequency, which is 133 cm^−1^ for Pb-Br-Pb stretch, based on previous report. The electron–phonon coupling strengths (Γ_1_) in Supplementary Fig. 15 were fitted to be 0.032 eV and 0.035 eV in PEACl:CsPbBr_3_(1:1) films with or without YCl_3_, respectively. Γ_0_ was reduced from 0.093 eV in PEACl:CsPbBr_3_(1:1) film to 0.086 eV in PEACl:CsPbBr_3_(1:1) film with YCl_3_.

*Density functional theory calculations* were performed using the VASP code^45,46^ with projector augmented-wave^47^ potentials. The PBE^48^ exchange-correlation functional and a kinetic energy cutoff of 300 eV were employed. Spin–orbit coupling was included in the calculations due to the heavy Pb atoms. To model the surface passivation, we built a CsPbBr_3_ slab with the top surface layer covered by [YCl_6_] octahedra. The cell size is 1.2 nm × 1.2 nm × 2.3 nm. A vacuum thickness of 15 Å was used to eliminate spurious periodic interactions between the slabs. The bottom two layers of [PbBr_6_] octahedra were fixed at their relaxed bulk positions, while all other atoms were relaxed with a force tolerance of 0.01 eV Å^−1^. A *Γ-*centered *k* mesh of 4 × 4× 1 was used to sample the Brillouin zone.

## Supplementary information

Supplementary Information

## Data Availability

The data that support the findings of this study are available from the corresponding author upon reasonable request. The source data underlying Figs. 1a, d,  2c-i,  3a-e, and 4a, c–f are provided as a Source Data.

## Source data

Source Data
