# Supplementary material for: Efficient sky-blue perovskite light-emitting diodes via photoluminescence enhancement
Source: Nat Commun. 2019 Dec 10;10:5633. doi: 10.1038/s41467-019-13580-w (PMC6904584; doi:10.1038/s41467-019-13580-w)
Supplement: Supplementary file 1 — Supplementary Information [file 41467_2019_13580_MOESM1_ESM.pdf]

Supplementary Information for

Efficient Sky-blue Perovskite Light-emitting Diodes via Photoluminescence Enhancement

Wang et.al

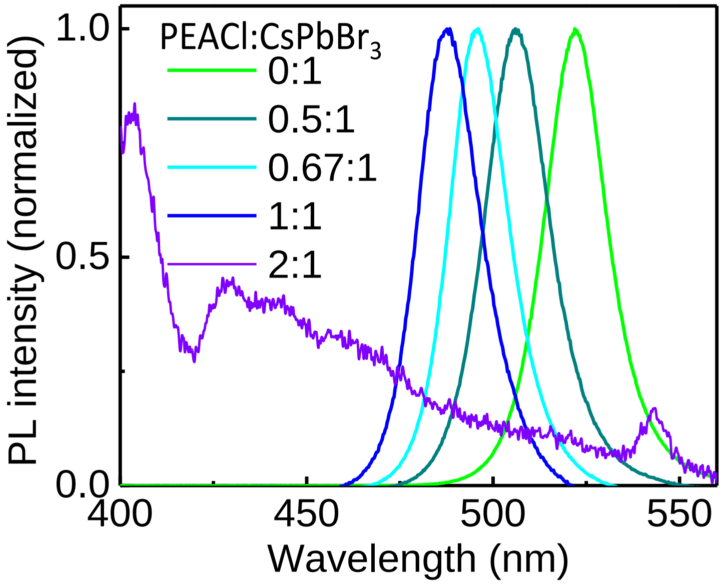

Supplementary Figure 1 PL spectra of perovskite films with different ratios of PEACl to $CsPbBr_3$

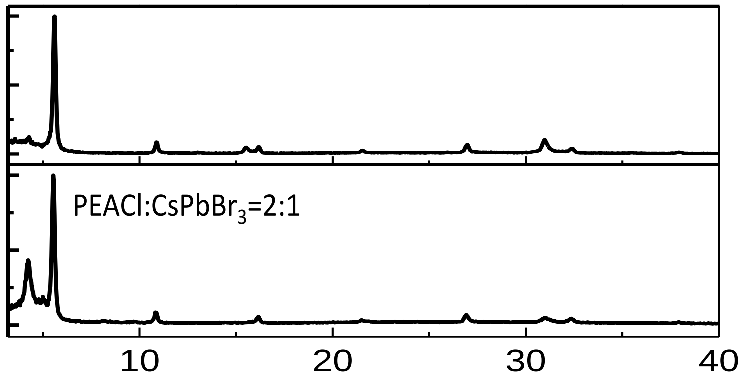

Supplementary Figure 2 XRD patterns of PEACl:$CsPbBr_3$ (1:1) and PEACl:$CsPbBr_3$ (2:1) films.

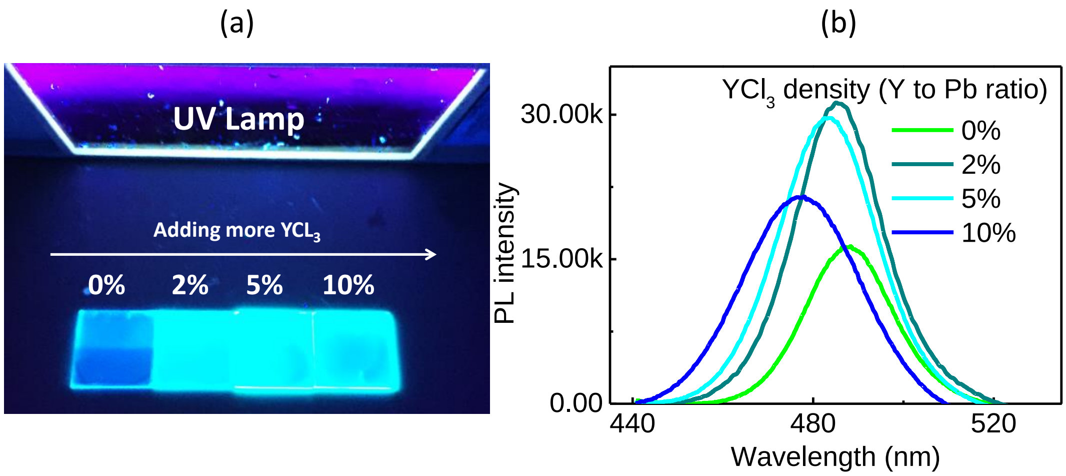

Supplementary Figure 3 A photograph of $CsPbBr_3$:PEACl (1:1) perovskite films with different ratios of YCl_3 under the excitation of a UV lamp. The percentage numbers in the figure represent the Y/Pb molar ratios in the precursor solutions. (b) PL spectra of $CsPbBr_3$:PEACl (1:1) perovskite films with different ratios of YCl_3. The PL peaks are at 487 nm, 485 nm, 483 nm, 477 nm for the films with 0%, 2%, 5%, 10% YCl_3, respectively.

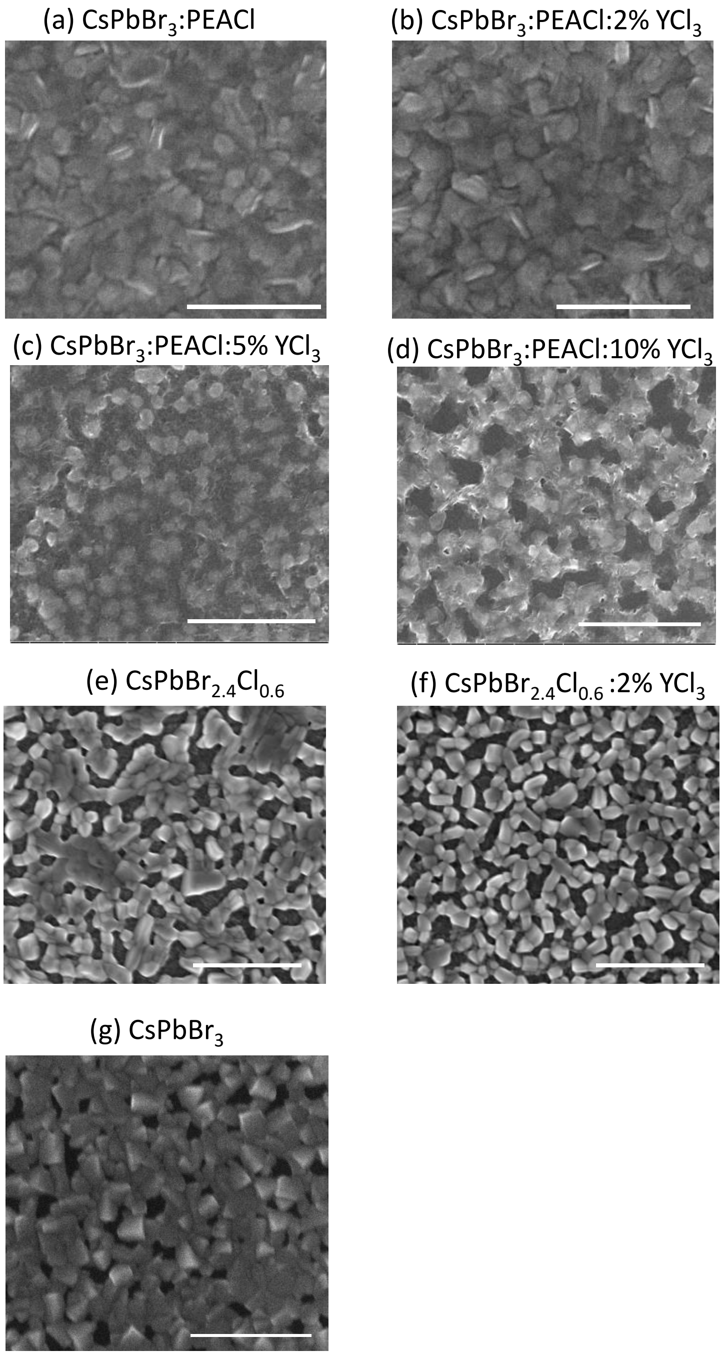

Supplementary Figure 4 SEM images of $CsPbBr_3$:PEACl (1:1) films without YCl_3 (a) and with 2% (b), 5% (c), 10% (d) YCl_3. SEM images of $CsPbBr_{2.4}Cl_{0.6}$ film (e) and $CsPbBr_{2.4}Cl_{0.6}$ film with 2% YCl_3 (f). (g) SEM image of $CsPbBr_3$ film. The scale bar in a-d is 500 nm. The scale bar in e-g is 1 μm.

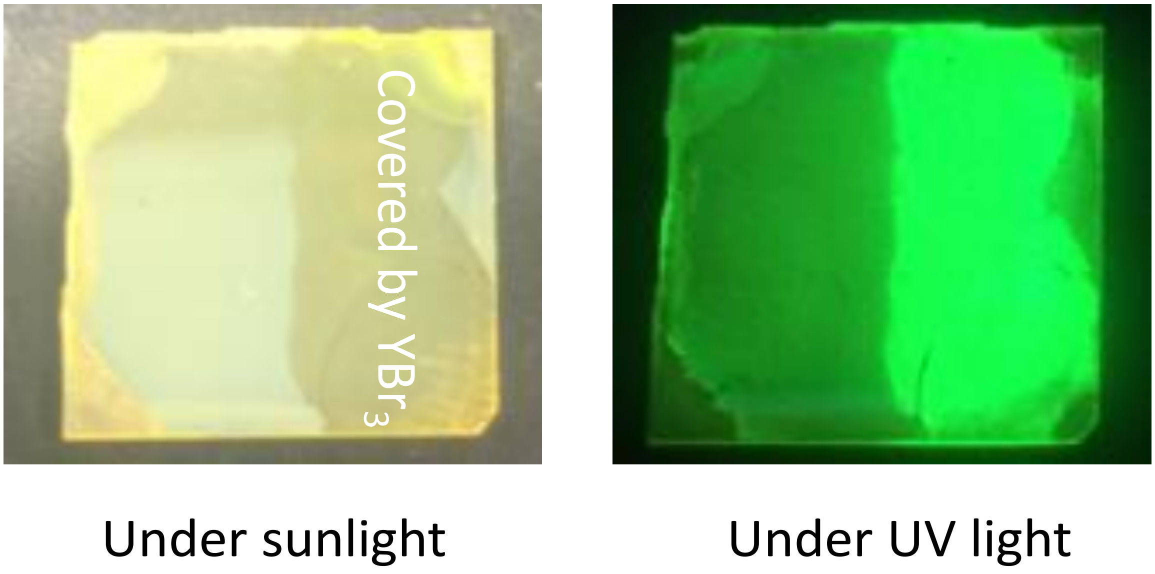

Supplementary Figure 5 Photos of a $CsPbBr_3/YBr_3$ film under sunlight (left) and under UV lamp (right). $CsPbBr_3$ film was firstly fabricated by spin coating on a glass substrate and annealed at 90 ℃ for 20 min. Then YBr_3 dissolved in IPA solvent was dropped to cover half area of the film and annealed at 90 ℃ for 20 min.

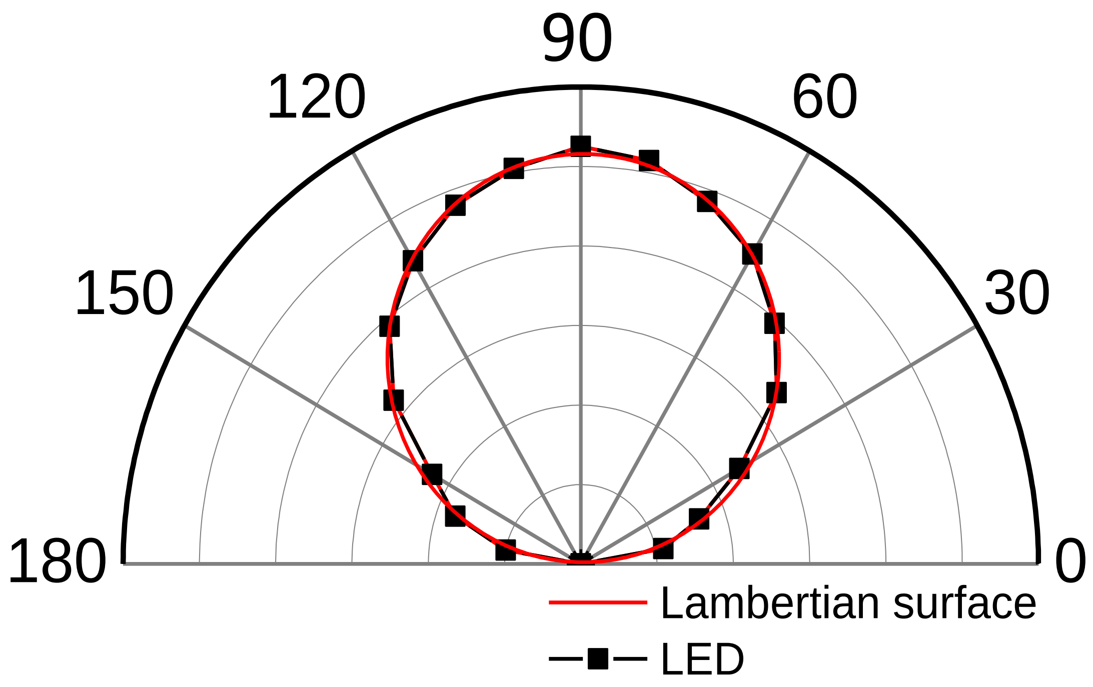

Supplementary Figure 6 Angular distribution of radiation intensity of a ~11% efficiency LED.

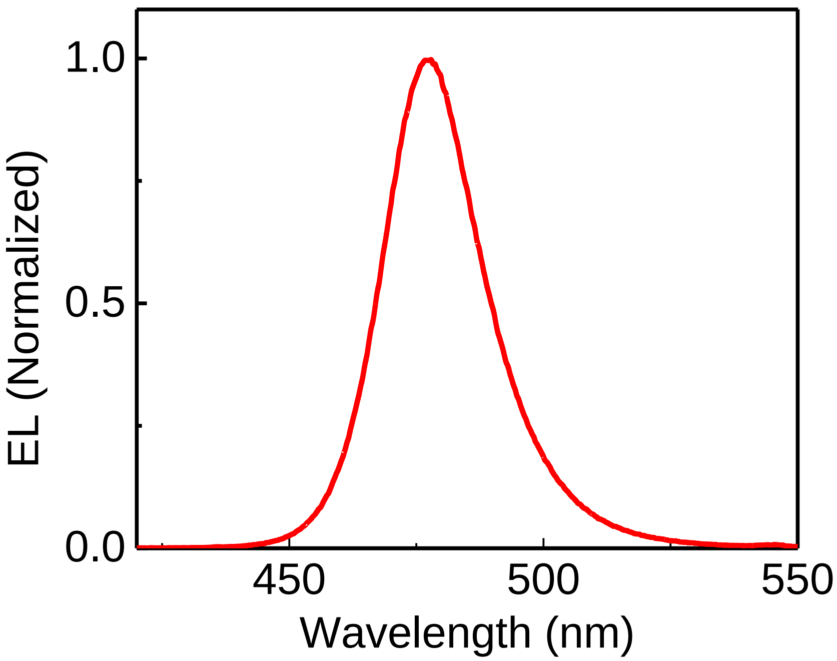

Supplementary Figure 7 EL spectrum of $CsPbBr_3$:PEACl (1:1) device with 10% YCl_3. The EL peak is at 477 nm.

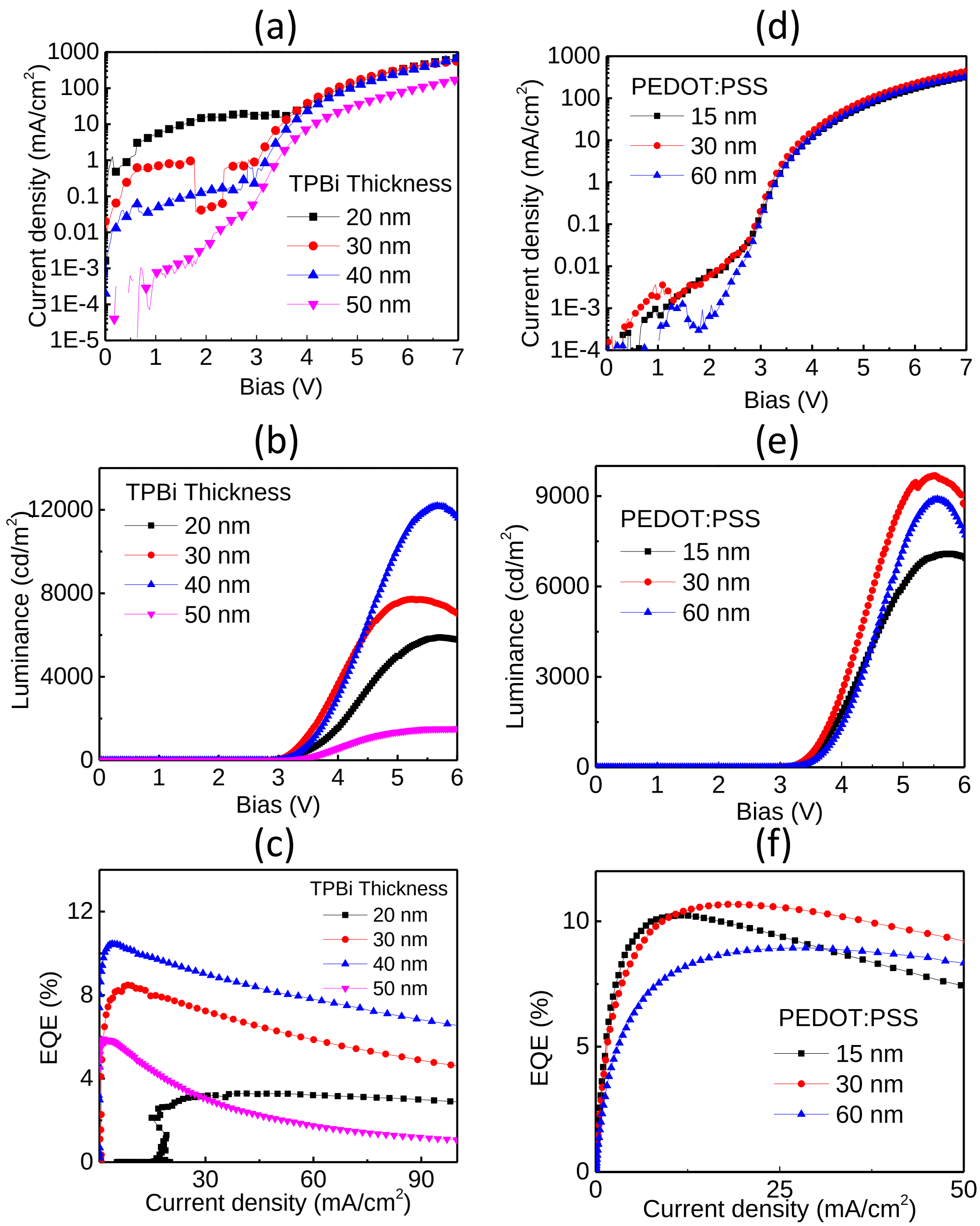

Supplementary Figure 8 Current density-bias (a) Luminance-bias (b), EQE-current density (c) curves of $CsPbBr_3$:PEACl:YCl_3 devices with different TPBi thicknesses. Current density-bias (d) Luminance-bias (e), EQE-current density (f) curves of $CsPbBr_3$:PEACl:YCl_3 devices with different PEDOT:PSS thicknesses. The molar ratio of $CsPbBr_3$:PEACl is 1:1 and Y to Pb ratio is 2%.

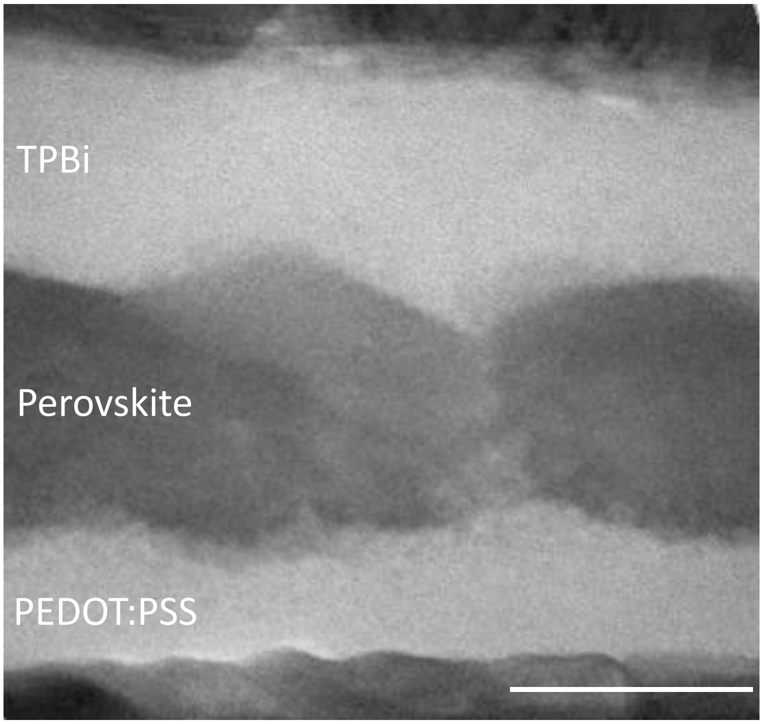

Supplementary Figure 9 TEM image of a device with perovskite layer thickness of ~50nm. The scale bar is 50 nm. The composition of perovskite layer is $CsPbBr_3$:PEACl (1:1) with 2% YCl_3

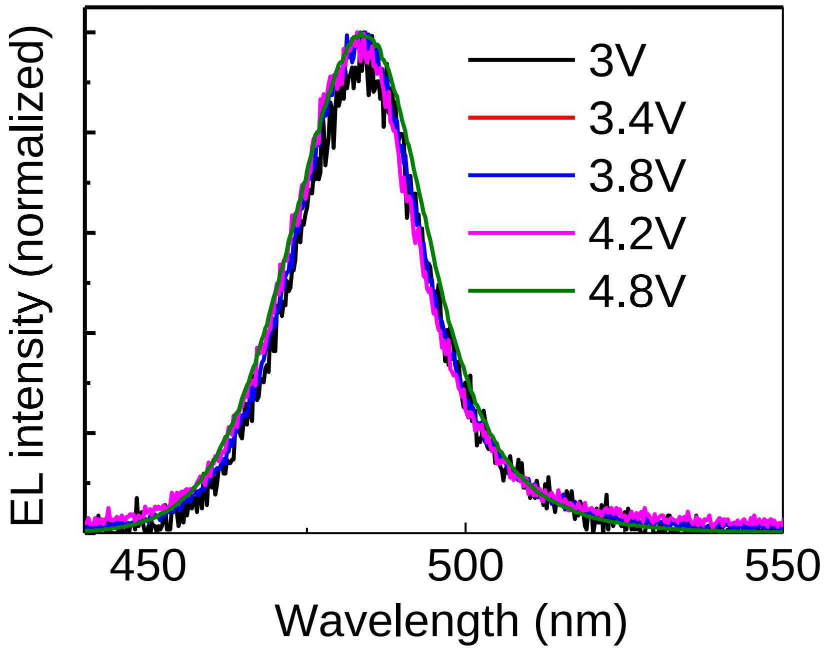

Supplementary Figure 10 EL spectra of a sky-blue LED under different biases

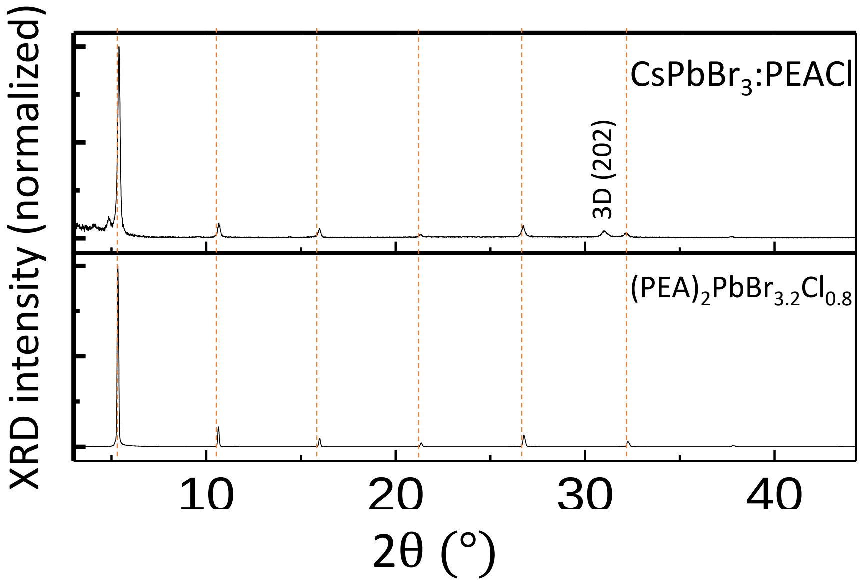

Supplementary Figure 11 XRD of CsPbBr3:PEACl (1:1) film and 2D $(PEA)_2PbBr_{3.2}Cl_{0.8}$. XRD peaks of n=1 phase in $CsPbBr_3$:PEACl (1:1) film are very close to those of 2D $(PEA)_2PbBr_{3.2}Cl_{0.8}$, as marked by the dashed lines.

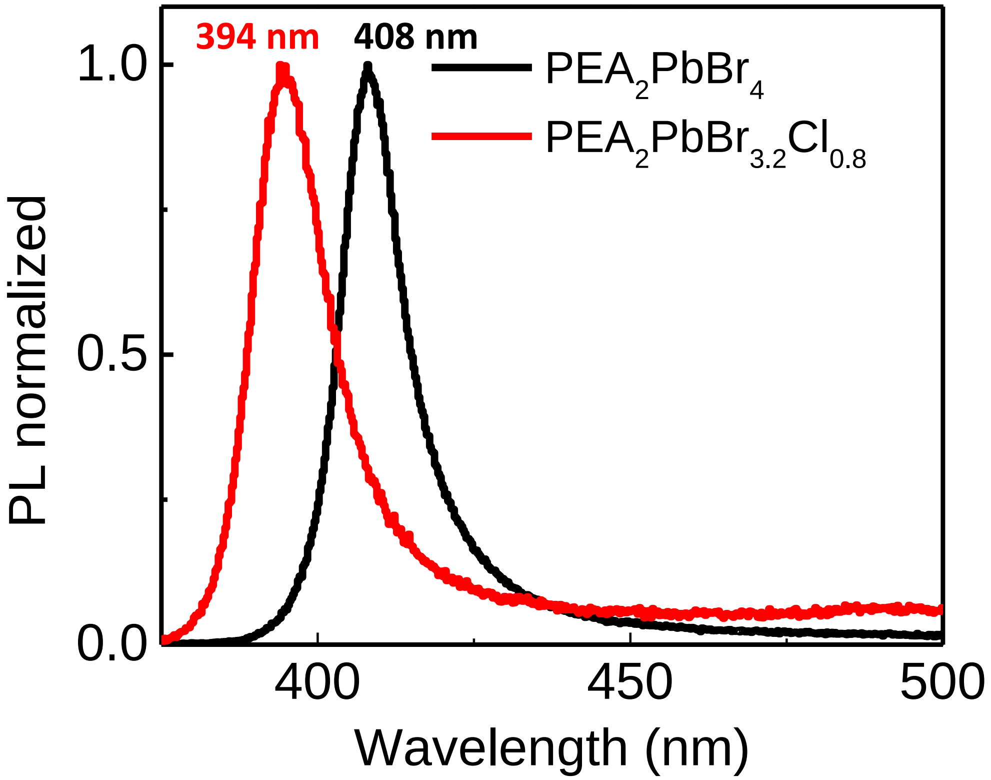

Supplementary Figure 12 PL spectra of $(PEA)_2PbBr_4$ and $(PEA)_2PbBr_{3.2}Cl_{0.8}$ films. The PL from n=1 phase in $CsPbBr_3$:PEACl (1:1) film is close to the PL of $(PEA)_2PbBr_{3.2}Cl_{0.8}$ film.

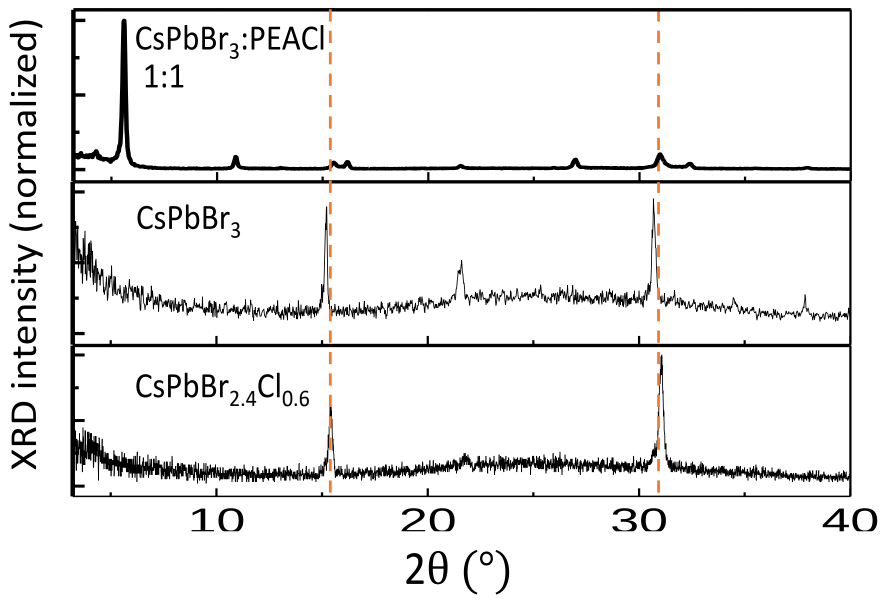

Supplementary Figure 13 XRD of CsPbBr3:PEACl (1:1), $CsPbBr_3$, $CsPbBr_{2.4}Cl_{0.6}$. The XRD peaks of 3D phase in CsPbBr3:PEACl (1:1) film is closer to $CsPbBr_{2.4}Cl_{0.6}$ film than $CsPbBr_3$ film

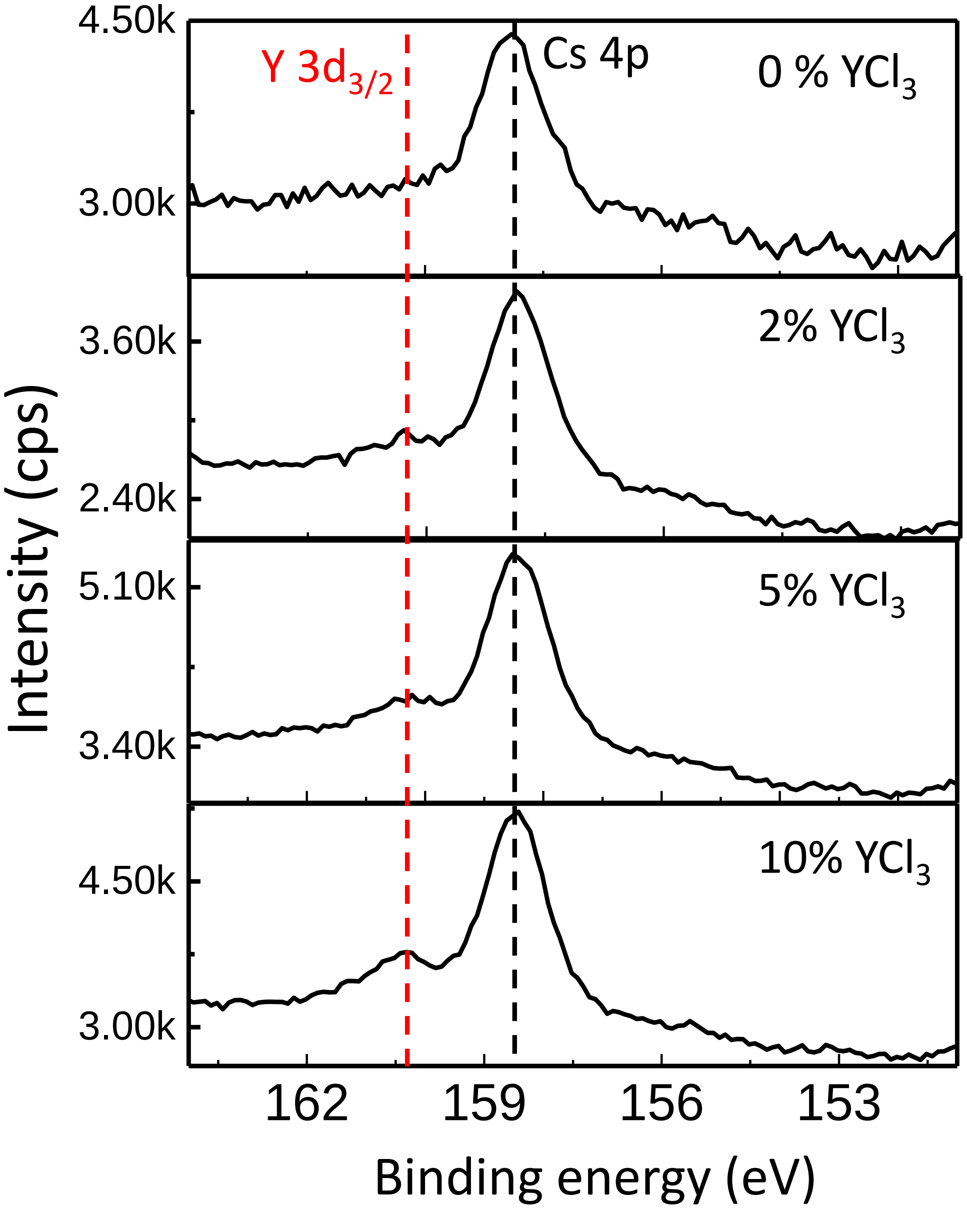

Supplementary Figure 14 XPS measurements of PEACl:$CsPbBr_3$ (1:1) films with different ratios of YCl_3. The Y $3d_{3/2}$ peak at 160.4 eV (red dash line) obviously increases with yttrium incorporation in the films.

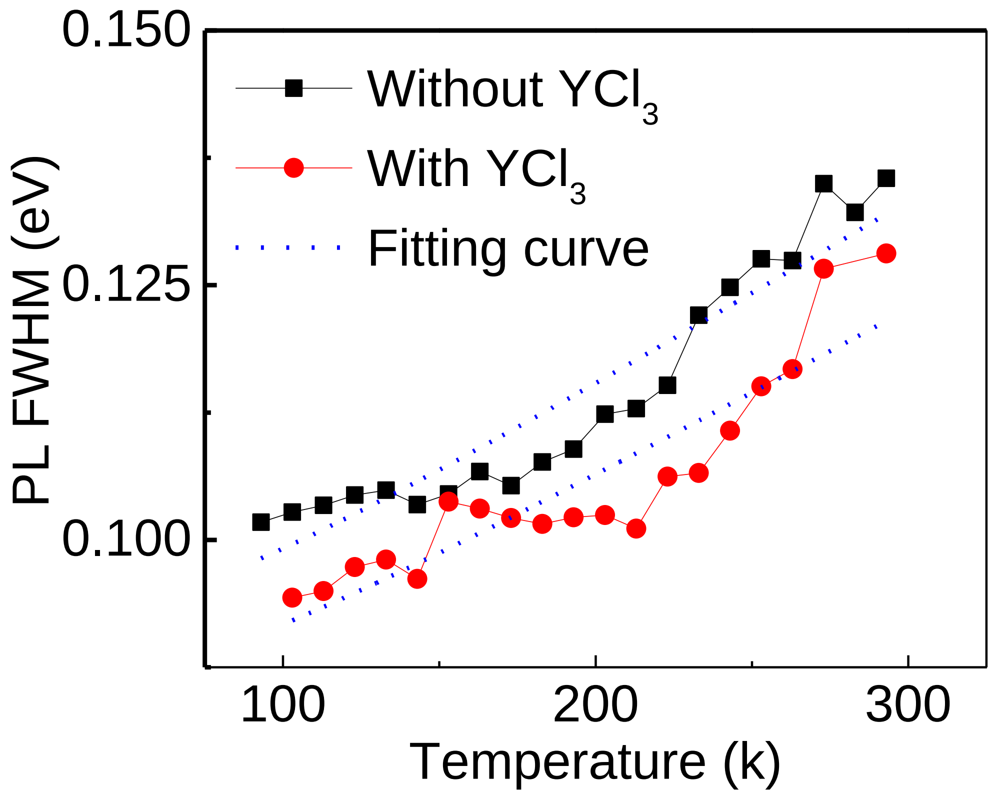

Supplementary Figure 15 PL FWHM of CsPbBr3:PEACl (1:1) with or without 2% YCl_3 under different temperatures. The curves were fitted by using the equation (3) described in the Methods of manuscript. The phonon-electron coupling strength (Γ_1) was fitted to be 0.032 eV and 0.035 eV in PEACl:$CsPbBr_3$(1:1) films with or without YCl_3, respectively. The temperature-independent term (Γ_0) was fitted to be 0.093 eV in PEACl:$CsPbBr_3$(1:1) film and 0.086 eV in PEACl:$CsPbBr_3$(1:1) film with 2% YCl_3.

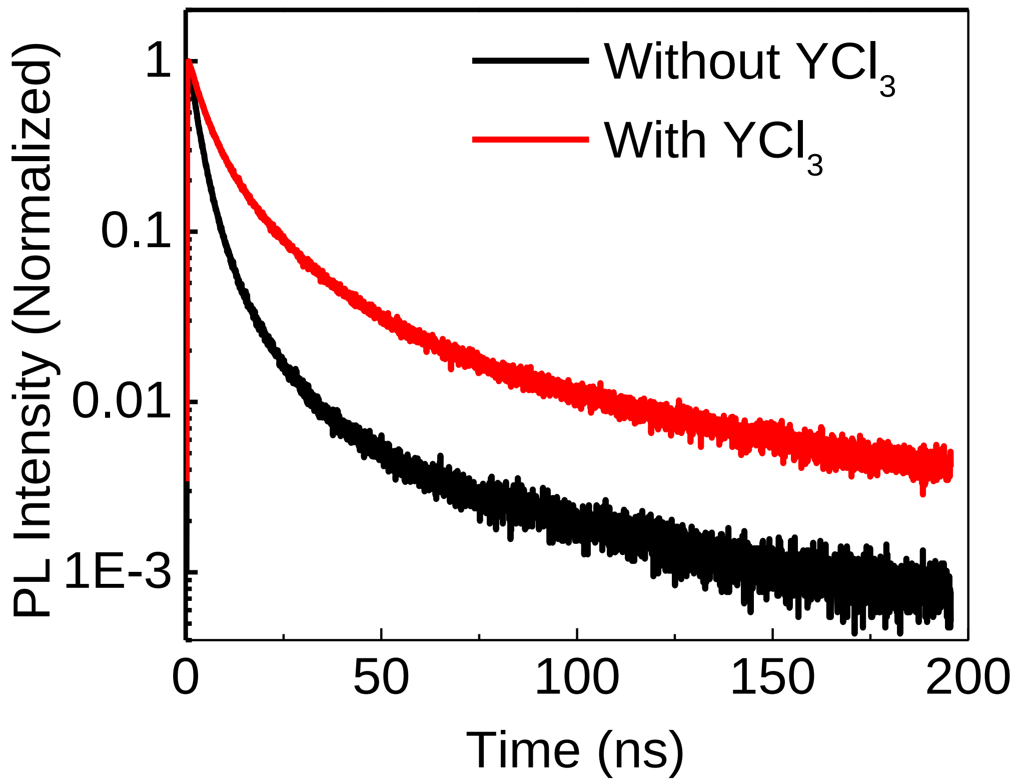

Supplementary Figure 16 Time-resolved photoluminescence decay of $CsPbBr_3$:PEACl (1:1) films with or without 2% YCl_3

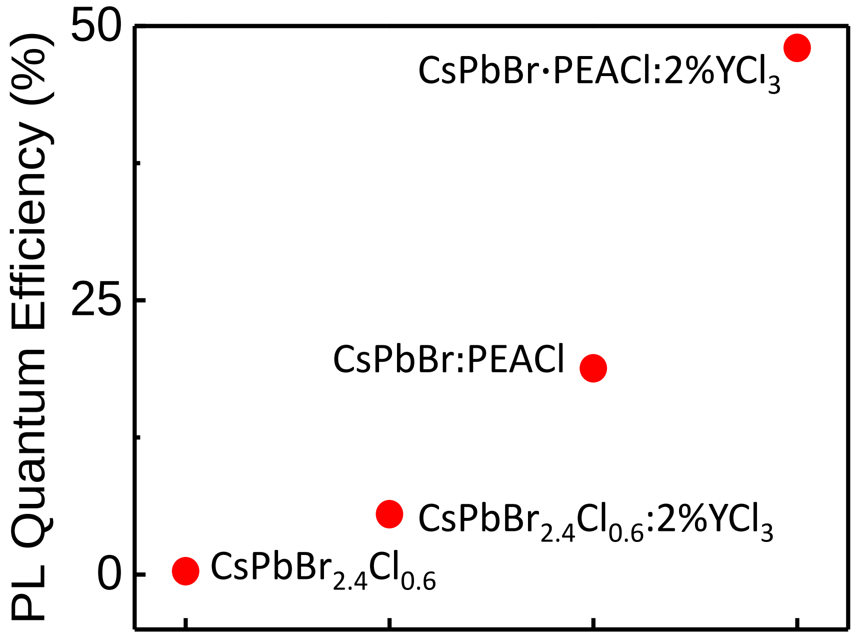

Supplementary Figure 17 PLQEs of perovskite films with different compositions. The measurement was performed by our collaborator in Xi’an Jiao Tong University in China.
